# Field trial to evaluate the effect of an intranasal respiratory vaccine protocol on bovine respiratory disease incidence and growth in a commercial calf rearing unit

**DOI:** 10.1186/s12917-020-02294-7

**Published:** 2020-03-04

**Authors:** Atte Sandelin, Heidi Härtel, Leena Seppä-Lassila, Liisa Kaartinen, Helena Rautala, Timo Soveri, Heli Simojoki

**Affiliations:** 1 Department of Production Animal Medicine, Faculty of Veterinary Medicine, University of Helsinki, Paroninkuja 20, 04920 Saarentaus, Helsinki, Finland; 2 HKScan Finland Oy, 50, 20521 Turku, PL Finland; 3 Finnish Food Authority Ruokavirasto, Mustialankatu 3, 00790 Helsinki, Finland

**Keywords:** Intranasal vaccination, Bovine respiratory disease, BRD, Morbidity, Mortality, Calf rearing unit

## Abstract

**Background:**

Bovine respiratory disease (BRD) continues to be great challenge in calf rearing units. The urgent need to decrease the use of antibiotics and increase animal welfare in beef production has forced us to introduce new preventive methods. Vaccinations could contribute to the solution, but the high incidence of BRD already at an early age has made it difficult to introduce suitable vaccination programs. Challenge studies have shown promising results in 3–14 day old calves vaccinated with intranasal BRD vaccine, but very few field trials are available to assess the efficacy of the intranasal vaccines in field conditions. We evaluated the effect of one dose of commercial intranasal vaccination on calf mortality, daily gain, and treatment incidence for BRD in one calf rearing unit. In total, 497 calves (mean age 19 days) were included in our study, 247 of which were vaccinated at the time of arrival to the unit and 250 served as negative controls (unvaccinated). Vaccinated and unvaccinated calves were situated in separate compartments until weaning. Daily gain, treatment incidence, and mortality were recorded until the calves were transported to the finishing unit, which averaged 154.5 days from arrival.

**Results:**

Average daily gain over the complete study period was 1151.9 g/day (SD 137.9) for the vaccinated calves and 1139.5 g/day (SD 135.9) for the unvaccinated calves. Intranasal vaccination combined with older arrival age (17 days or older) resulted in a higher daily gain (47.8 g/day) compared with unvaccinated calves (coef. 0.0478, *p* = 0.003). This association was not recorded in calves that were younger than 17 days upon arrival. Intranasal vaccination was not significantly associated either with mortality (OR 0.976, *p* = 0.968) or treatment incidence for BRD (OR 1.341, *p* = 0.120). In total, six vaccinated calves (2.43%) and six unvaccinated calves (2.40%) died during the study period.

**Conclusions:**

Vaccinating arriving calves with intranasal vaccine in the calf rearing unit did not decrease the mortality or treatment incidence for BRD, but it significantly improved the weight gain in calves transported to the unit at the age of 17 days or older.

## Background

Bovine respiratory disease (BRD) is the major cause of increased morbidity and mortality in young calves [1–3]. BRD causes substantial economic losses for the cattle industry, and can also be seen as an animal welfare issue [4]. Several studies have demonstrated the negative economic impact of increased mortality, treatment costs, and reduced growth caused by BRD [5–7]. Moreover, high morbidity rates lead to increased use of antibiotics, which is commonly associated with the emergence of antimicrobial resistance [8]. Together with veal calf operating units in Europe, calf rearing operations in Finland consume reasonably high levels of antibiotics [2, 9, 10]. A recent study published in the Netherlands showed that antimicrobial consumption rates in veal calf farms was several times higher compared with pig and broiler farms [9]. Public concern of animal welfare and emergence of antibiotic resistance puts pressure on the industry to find new preventive methods for improving animal health, and thus reduce the use of antibiotics in the beef production chain [11].

BRD has a multifactorial origin, and can be caused by several different pathogens either alone or combined [12, 13]. Earlier studies have shown that BRD incidence is highly dependent on environmental, individual, and farm specific factors [1, 5]. Respiratory syncytial virus, corona virus, parainfluenza type 3 virus, *Pasteurella multocida, Histophilus somni, Mannheimia haemolytica*, and *Ureaplasma diversum* are common respiratory pathogens around the world, and also the most frequently found pathogens in cattle respiratory samples in Finland [12–14]. *Mycoplasma bovis* was first isolated from a Finnish cattle respiratory sample in 2012. Currently, Finland is free from infectious bovine rhinotracheitis and bovine viral diarrhea [14].

Beef production in Finland is mainly based on rearing bull calves born in dairy farms on separate finishing farms specialized in beef production. These bull calves are commonly either pure dairy breeds or dairy-beef crosses. They are transported to separate rearing units at 10–30 days of age. Unlike veal calf production in central Europe and North America, calves in Finland are not slaughtered until the age of 18–20 months. Approximately two thirds of dairy bull calves are first transported to specialized calf rearing units where they are reared for 4–6 months before transportation to the specialized finishing farms. The remaining calves are either transported to the finishing farms directly after weaning, or to the integrated beef production farms specialized for rearing unweaned calves to the slaughter age. Prophylactic use of antibiotics is very rare in Finland.

Unlike in many other countries, vaccinations against BRD are not commonly used in Finland. The growing size of calf rearing units and rising incidence of BRD has forced us to introduce new preventive methods. Vaccinating calves more frequently against common respiratory pathogens in the future could be one part of the solution. Since morbidity and mortality rates are usually highest in the first weeks after arrival to the calf rearing units [3, 15] and maternal antibodies are known to have an inhibitory effect on parenterally administered vaccinations [16, 17], developing an effective vaccination program for young calves has been problematic. This blocking effect has increased the interest in introducing intranasally administrable vaccinations. Hill and others [18] showed that 3–8 day old Holstein calves are capable of developing mucosal immune response after intranasal vaccination, even when maternal antibodies are present. Other studies of intranasal vaccinations have also shown promising results in young calves (3–14 days old) vaccinated against common respiratory pathogens [19–21]. The short duration of immunity after one dose of intranasal vaccination may set a requirement for subsequent booster vaccines [19, 22]. Even though intranasal vaccinations have not reduced the incidence of BRD in some studies, pathological changes in lungs have been reduced [22, 23]. As calves are transported to the rearing unit from numerous farms, it would be more achievable to start vaccinating calves at the time of arrival to the rearing unit, even though the best vaccine effect would require vaccination already before transportation. Despite the increased interest in intranasal vaccinations, very few clinical trials are available to prove their efficacy in field conditions.

The primary objective of this randomized field trial was to evaluate the effect of a single dose of commercial intranasal vaccination on treatment incidence for BRD, daily gain, and calf mortality in one commercial calf rearing unit.

## Results

### Descriptive statistics

Upon arrival, 32.0% (*n* = 79) of the vaccinated calves and 30.4% (*n* = 76) of the unvaccinated calves were assessed to have BRD (Table 1). The serum immunoglobulin G (IgG) concentration at the time of arrival was below the reference value (10 mg/ml) in 51.8% (*n* = 128) of the vaccinated calves and 57.6% (*n* = 144) of the control calves (54.7% (*n* = 272) of all calves). Overall, 23.3% (*n* = 116) of the calves had serum amyloid A (SAA) values higher than the reference value (178 μg/ml) [24]. The SAA reference value was exceeded in 26.7% (*n* = 66) and 20% (*n* = 50) of the vaccinated and unvaccinated calves, respectively. Differences in measured variables were not statistically significant between vaccinated and unvaccinated calves.

**Table 1:** Descriptive statistics of the 497 calves at the time of arrival

	Total ± SD (min.-max.)	Vaccinated ± SD (min.-max.)	Unvaccinated ± SD (min.-max.)
n	497	247	250
Number of compartments	14	7	7
Sex: Male/female	467/30	231/16	236/14
Mean age at arrival/days	17 ± 4.9 (10–45)	16.7 ± 5.2 (10–45)	17.2 ± 4.6 (10–34)
Mean weight at arrival/kg	56.6 ± 8.8 (40–92)	56.8 ± 9.2 (40–92)	56.5 ± 8.4 (40–92)
Mean serum IgG ^a^	10.2 ± 5.8 (0.7–33.9)	10.5 ± 5.9 (0.7–33.9)	9.8 ± 5.6 (0.7–27.7)
Mean serum SAA^a^	123.4 ± 81.1 (9.2–609.6)	127.1 ± 81.4 (9.2–436.8)	119.6 ± 80.7 (9.4–609.6)
Clinical condition ^a b^	2.7 ± 2.0 (0–10)	2.7 ± 2.1 (0–10)	2.8 ± 2.0 (0–10)
BRD diagnosis ^a b^	155	79	76
Breeds (n):			
Ayrshire	191	87	104
Holstein	180	89	91
Finnish cattle	2	1	1
Dairy beef crosses	124	70	54

*SD* standard deviation, *SAA* Serum amyloid A, *BRD* Bovine respiratory disease,

^a^ = Observed/measured at the first clinical examination, ^b^ = See the text for detailed description

During the study period, 318 (64%) calves received at least one course of antibiotics. Specifically, 204 calves received one, 102 calves received two, and 12 calves received three antibiotic courses. Only three calves had antibiotics administered three times for the same diagnosis. In total, 51% of the antibiotic courses were targeted against BRD, 22.6% against interdigital phlegmon, 17.2% against unknown fever and 9.3% against other infections (umbilical inflammation, ear inflammation, arthritis). Only two types of antibiotics were used during the study period. Parenteral oxytetracycline was used in 68.6% of the courses, and parenteral benzylpenicillin in 31.4%. A non-steroidal anti-inflammatory drug was added to every antimicrobial course. On average, time to first medication was 2.1 days longer in vaccinated calves compared with unvaccinated calves (Table 2). The difference in time to first medication was not statistically significant between groups.

**Table 2:** Descriptive statistics of the 497 calves over the complete study period (average 154.5 days)

	Total ± SD (min.-max.)	Vaccinated ± SD (min.-max.)	Unvaccinated ± SD (min.-max.)
n	497	247	250
Average weight at weaning (kg)	83.4 ± 12.0 (40.5–126)	83.9 ± 12.6 (40.5–122)	82.8 ± 11.3 (52.5–126)
Average weight at the end of the study (kg)	233.7 ± 25.6 (126–310)	233.4 ± 26.2 (146–310)	233.9 ± 25.1 (126–302)
Daily gain, milk-feeding period (g/day)	630.7 ± 176.6 ((− 40)-1160)	641.7 ± 184.6 ((− 40)-1070)	619.6 ± 168.1 (150–1160)
Daily gain, complete study period (g/day)	1145.7 ± 136.9 (500–1520)	1151.9 ± 137.9 (670–1520)	1139.5 ± 135.9 (500–1460)
n (enrolled < 17 days of age)	261	139	122
Daily gain, (g/day)	1119.7 ± 131.0 (670–1460)	1109.3 ± 134.7 (670–1410)	1131.5 ± 126.2 (780–1460)
n (enrolled ≥17 days of age)	224	102	122
Daily gain, (g/day)	1176.0 ± 137.6 (500–1520) ^a^	1209.9 ± 120.3 (880–1520)	1147.6 ± 145.0 (500–1460)
Clinical condition (2nd clin. exam) ^b^	2.7 ± 1.9 (0–9)	2.7 ± 2 (0–8)	2.8 ± 1.8 (0–9)
BRD-diagnosis (2nd clin. exam) ^b^	206 ^a^	91	115
Antimicrobial treatments for	443	233	210
BRD	226	120	106
interdigital phlegmon	100	55	45
other inflammation	41	21	20
unknown fever	76	37	39
Oxytetracycline courses	304	157	147
Benzylpenicillin courses	139	76	63
Time to first medication (days)	20.7 ± 18.6 (0–93)	21.7 ± 19.2 (0–81)	19.6 ± 17.9 (0–93)
Premature death	12	6	6

*SD* standard deviation, *BRD* Bovine respiratory disease,

^a^ = Variable significantly different between vaccination groups (*p* < 0.05), ^b^ = See the text for detailed description

### Average daily gain before weaning

Weight of the vaccinated calves was not significantly different than unvaccinated calves when measured at weaning (*p* = 0.328) (Table 2). In the regression model, vaccination was not significantly associated with daily gain before weaning (coef. 0.028, *p* = 0.057). Female sex of the calf (coef. 0.073, *p* = 0.033), older age at arrival to the unit (coef. 0.185, *p* = 0.000), and positive diagnosis of BRD at first clinical examination (coef. 0.040, *p* = 0.012) were associated with better growth in the milk-feeding period. Other independent factors in the model were breed and serum SAA concentration. In total six calves were excluded from the model (one due to missing weight data and five due to untimely death during the milk-feeding period).

### Average daily gain during the complete study period

**Fig. 1:**
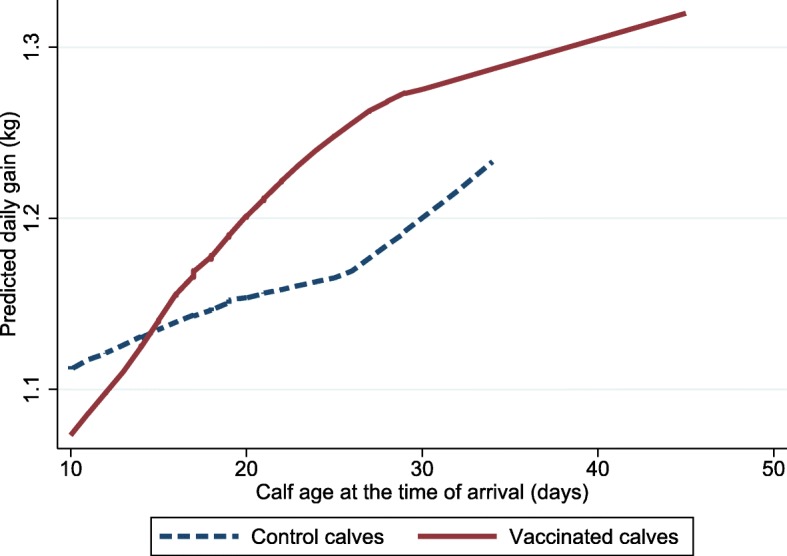
Interaction between intranasal vaccination and calves’ age at arrival to the calf rearing unit. In total, 247 calves were vaccinated and 250 calves served as negative controls (unvaccinated). Predicted values for daily gain during the whole study period are estimates from the regression model

Average daily gain of the calves from arrival to the unit until the end of the study period is presented in Table 2. During the complete study period, vaccinated calves gained an average of 12.3 g/day more than unvaccinated calves. In the first final model, an interaction between vaccination and age at time of arrival was detected (Fig. 1). Thereafter, two different mixed regression models were generated. One for calves younger than 17 days (*n* = 261) and another for calves 17 days or older (*n* = 224) at the time of arrival to the unit. Vaccinated calves had significantly better growth compared with unvaccinated control calves (coef. 0.048, *p* = 0.003) in the model for calves 17 days or older at the time of arrival. Other independent factors in the model positively associated with growth were weight of the calf at arrival (coef. 0.004, *p* < 0.001) and beef breed (coef. 0.051, *p* = 0.030). Female sex (coef. -0.103, *p* = 0.001) negatively affected the growth. In the model for calves younger than 17 days at the time of arrival, vaccination did not have a significant effect on growth (coef. -0.017, *p* = 0.239). The only factor positively affecting growth in younger calves was higher weight of the calf at the time of arrival to the unit (coef. 0.005, *p* < 0.001). In total 12 calves were excluded from the models due to untimely death during the study period.

### BRD diagnosis at the time of the second clinical examination

Vaccinated calves had lower odds ratios (OR) for BRD diagnosis during the second clinical examination compared with unvaccinated calves (OR = 0.639, *p* = 0.022). BRD was diagnosed in 37.0% of vaccinated and 46.8% of unvaccinated calves. Other variables in the logistic regression model were age at arrival to the unit (OR = 1.627, *p* = 0.177), BRD diagnosis (OR = 1.510, *p* = 0.107), and clinical score (OR = 1.048, *p* = 0.418) at the first clinical examination. None of the other variables had statistically significant effects on the dependent variable. In total five calves were excluded from the model due to untimely death during the milk-feeding period.

### Treatments for BRD

In total, 34.2% of all calves were treated once for BRD (31.2% of unvaccinated, 37.3% of vaccinated calves). 5% were treated two times (4.4% unvaccinated, 5.7% vaccinated) and 0.4% were treated three times (0.8% unvaccinated, 0.0% vaccinated) for BRD during the study period. From vaccinated calves, 42.9% were treated for BRD at least once during the study period; for the unvaccinated calves, the total was 36.4%. However, the difference between vaccinated and unvaccinated calves was not statistically significant (OR = 1.341, *p* = 0.120) according to the mixed regression model that tested the probability of the calf to be treated against BRD at least once during the study period. Other factors preserved in the model were serum IgG and SAA levels at arrival to the unit (OR = 0.968, *p* = 0.058; OR = 1.214, *p* = 0.147), calf weight at arrival to the unit (OR = 0.984, *p* = 0.154), BRD diagnosis (OR = 1.337, *p* = 0.247), and clinical condition at arrival to the unit (OR = 1.071, *p* = 0.224). None of these factors had statistically significant effects on the chance to be treated for BRD. All the study calves (*n* = 497) were included in the model.

### Mortality

Mortality during the study period was 2.41%. Six (2.43%) vaccinated and six (2.40%) unvaccinated calves died during the study period. Three whole calves and two lung-heart packages were sent to necropsy to determine the cause of death. Pneumonia was determined as the cause of death in two control calves, polyarticular arthritis combined with omphalitis was the cause of death in one vaccinated calf, and unidentified gastrointestinal disease was the cause of death in one control calf. The cause of death could not be determined for one necropsied calf. The remaining seven dead calves were not necropsied. The mean time spent in the unit before their untimely death was 83.3 days (SD 51.2, range = 28–180). The difference in mortality between vaccinated and unvaccinated calves was not statistically significant in the logistic mixed regression model (OR = 0.870, *p* = 0.817). Only one other factor preserved in the model was the number of antimicrobial treatments in addition to the confounding factors of sex and breed. The higher number of antimicrobial treatments during the rearing period significantly increased the odds that the calf would die during the study period (OR = 2.645, *p* = 0.006). All the study calves (*n* = 497) were included in the model.

## Discussion

In this study, BRD morbidity, average daily gain, and mortality of the calves vaccinated with intranasal BRD vaccine (Rispoval RS + Pi3 Intranasal, Zoetis) were compared with unvaccinated calves in one commercial calf rearing unit in Finland. Vaccination combined with older arrival age (17 days or older) resulted in significantly greater daily gain (on average 47.8 g) compared with unvaccinated calves. This difference was not recorded in calves that arrived when they were younger than 17 days. In our study, all the vaccinated calves were vaccinated one to three days after arrival. This finding suggests that younger calves are not capable of responding to the vaccination as effectively as older calves. Further studies are needed to determine if this is a result of the younger calves having an immature immune system, or inhibitory effects of maternal antibodies. Vaccination also showed a trend towards increasing daily gain during the milk-feeding period independent of age at the time of arrival. Additionally, vaccination lowered the odds ratio for BRD diagnosis at the second clinical examination.

The efficacy of intranasal vaccinations has been demonstrated in many experimental challenge studies, but very few clinical field trials are available [19, 20, 25, 26]. Ollivett and others [23] conducted a field trial in three dairy herds, where they intranasally vaccinated young calves for bovine rhinotracheitis, parainfluenza-3 virus and respiratory syncytial virus. The current study was conducted in a calf rearing unit, and it differed from the challenge studies in several ways. In our study, maternal antibody levels in calf serum displayed variation at the time of vaccination, and calves were naturally exposed to a wide range of pathogens. We knew from earlier studies and reports that bovine respiratory syncytial virus and parainfluenza-3 virus are rather common pathogens in Finnish cattle, thus we were quite confident that these pathogens would be present in the large rearing unit used in our study [12, 14, 27]. Here, we studied the efficacy of the vaccination under field conditions, which have probably also led to BRD cases caused by pathogens other than those included in the vaccine. The outcomes examined in our study were daily gain before weaning, daily gain during the complete study period, BRD diagnosis during the second clinical examination (at the time of weaning), number of antimicrobial treatments for BRD, and mortality during the study period.

Manufacturers recommend to vaccinate calves already ten days before a period of stress or high infection risk such as re-grouping or transportation, in order to achieve an optimal result. In practice, this is difficult to implement because the calves often have multiple origins, and dairy farmers are rarely motivated to vaccinate calves that are shortly leaving the farms. A more feasible practice could be to vaccinate calves at the time of arrival to the calf rearing unit (or immediately after), as we did in our study. We were aware that this may lead to decreased efficacy because the calves might get infected already before the vaccination or before the immunity has been successfully developed. According to our knowledge, there are no published efficacy studies of a similar intranasal vaccination protocol in the prevention of BRD in commercial calf rearing units. Nevertheless, the practice is commonly used in some European countries [28].

In our study, farm personnel used different overalls and boots in all compartments for the first ten days following vaccination to avoid the contamination of unvaccinated calves from potential nasal shedding by vaccinated calves. According to the vaccine manufacturer’s data, the shedding of BRSV and PI-3 V is possible up to 11 and 7 days after vaccination, respectively. Some studies have reported even longer shedding times [29]. According to this, risk of spreading vaccine viruses from vaccinated calves to unvaccinated calves via contaminated overalls or boots is possible even after ten days post vaccination. The authors are aware of this risk but it is considered minimal.

BRD diagnosis at the first clinical examination was associated with increased growth in the milk-feeding period. This positive association is hard to explain. The calf transportation system in Finland is based on the rule that only healthy animals are accepted for transportation. A decision is made by the dairy farmer and educated driver, but it is still a subjective decision and mild signs of a disease can be easily missed when a calf is otherwise apparently healthy. Bigger calves and those with higher growth potential are probably more likely to be transported despite the sickness, whereas small, older, and lower growth potential calves might be more readily excluded. This might lead to a situation where slightly ill calves with high growth potential are over-represented in the study population at the time of arrival.

Our BRD diagnosis was based on the scoring system used by Love and colleagues [30]; the only difference was that we used stethoscope auscultation rather than visual inspection to evaluate respiratory sounds, as was done in the original scoring system. The assessment of abnormal respiration based on audio sounds rather visual cues might have led to our finding of higher scores compared with the original scoring system. We also used a clinical score to describe the overall health of the calf at the time of clinical examination. Specifically, this was calculated by summing up the findings of the clinical examination. To our knowledge, this method is not based on any scoring system. Nevertheless, we considered it useful to account for disease conditions other than respiratory diseases when assessing the health of calves.

Vaccination was the only factor to significantly decrease the risk of BRD diagnosis in the second clinical examination. Using ultrasonography for the more accurate detection of lung lesions would have given more information on the actual status of the lungs. In a recent clinical trial, the intranasal vaccination lowered the odds of lung consolidation compared with unvaccinated calves, even though overall treatment for BRD was not reduced [23]. A similar effect might explain why the number of treatments against BRD was not reduced in the current study, even though the diagnosed BRD cases in the second clinical examination were reduced. Thompson and others [6] showed the association between lung lesions and decreased daily gain in slaughtered feedlot cattle. In the same study, they also demonstrated the existence of subclinical BRD by showing that lung lesions were presented in 38.5% of the animals never treated for BRD. This could also explain the enhanced daily gain in vaccinated calves in our study, even though the treatments for BRD were not reduced. Further studies are needed to prove this theory.

There was a trend towards high serum IgG at the time of arrival reducing the probability of the calf being treated for BRD. A similar association between low serum IgG levels and higher morbidity has also been presented in previous studies [31, 32]. To the best of our knowledge, there are no studied reference values for serum IgG levels for calves 2–4 weeks old. For two day old calves, 10 mg/ml is commonly used as the minimum level for successful passive transfer, but earlier studies have shown that the amount of IgG in serum decreases slowly after birth, with a half-life of 28.5 days [33]. In the current study, 44.1% of the calves with low serum IgG (< 10 mg/ml on arrival; mean age 17 days) were treated at least once against BRD, compared with 34.2% of the calves with high serum IgG (> 10 mg/ml at arrival, mean age 17 days). This supports earlier findings that have demonstrated how successful passive transfer of maternal antibodies is important for the health of the calf [34]. Of the arriving calves, 23.3% had serum SAA higher than the reference value < 178 mg/ml suggested by Seppä-Lassila and colleagues [24]. Calves with higher serum SAA concentration gained weight slower than calves with lower concentrations, as also reported in earlier studies [2]. Elevated concentrations of serum SAA may be normal in calves younger than two weeks old, or they may be a reflection of some existing inflammation already present at the time of arrival [24]. According to clinical examination data, 12.6% of the arriving calves suffered either healing or acute inflammation of the umbilicus.

The results of this study are promising even they cannot be adapted directly to all calf rearing units due to differences in pathogen profiles. The large number of different origin farms may even cause the pathogen profile variation between the consecutive batches of arriving calves. According to the results of this study, it would be worth considering transporting slightly older calves to the calf rearing units where the intranasal vaccination is used.

## Conclusions

Administration of a single intranasal dose of a commercially available vaccination significantly increased daily gain in calves that arrived at the calf rearing unit at the age of 17 days or older. Vaccination did not reduce the number of calves medicated for BRD, but it lower the incidence of diagnosed BRD at the time of weaning. A higher number of antimicrobial treatments during the rearing period significantly increased the odds of premature death.

## Methods

### Study population and study design

The randomized field trial was designed to test the efficacy of a single dose of commercial intranasal vaccine in one calf rearing unit. During the study, 247 calves were vaccinated at the time of arrival, and 250 calves served as negative controls (unvaccinated). Clinical examination and sample collection were carried out on all calves at two times (at arrival and weaning). Study calves were vaccinated during the first clinical examination. The study period started on the day of first clinical examination and lasted until the day when calves were transported to the finishing unit (average 154.5 days, SD 4.7). Daily gain was calculated during the period from arrival to weaning (milk-feeding period), and again from arrival to transportation to finishing unit (complete study period). Medications were recorded for the whole study period. Blood samples were analyzed to detect possible low serum IgG levels and high SAA levels at the time of enrolment. Normal operation cycle of the calf rearing unit remained throughout the study period and after study calves were transported to the feedlot for finishing cattle as usual. None of the calves were euthanized because of the study. Study design was approved in advance by the Animal Experimental Board of the Regional State Administrative Agency in Finland (ref. number: ESAVI/9788/04.10.07/2016).

The study was carried out in four similar milk-feeding compartments of one privately owned calf rearing unit located in southwestern Finland, between January 2017 and January 2018. The entire unit was comprised of six separate milk-feeding compartments and two larger separate uninsulated compartments for older calves. The unit was operated as an all-in all-out system, such that all six milk-feeding compartments were emptied, washed and disinfected prior to every arrival of new calves. The unit was confirmed to be free of *Mycoplasma bovis*. A milk replacer (Primo powermix, Suomen Rehu Oy, Finland) was dispensed by an automatic milk-feeding system (in total 33 kg/calf during the milk-feeding period), and all the calves had free entry to silage and concentrates (Primo Kasvatus 1, Suomen Rehu Oy, Finland). Each compartment contained one large pen with a solid floor feeding area and peat/straw bedding on the resting area. All compartments were insulated and had separate ventilation systems based on automatic exhaust fans. After 43 days of milk-feeding, calves were gradually weaned and then merged with the calves from the other compartments into one larger uninsulated compartment. Unlimited mixed feed was supplemented with 2 kg of grated barley/rapeseed mixture was offered to all calves daily from three weeks after weaning until the end of the study period (total concentrate percent around 55).

All calves used in this study (*n* = 497) were transported to the unit in seven batches (average 71 calves per batch, range = 68–76). All four similar milk-feeding compartments (average 36 calves each) were refilled every two months over a two-week time period. This cycle repeated four times, except in the last cycle (seventh batch) when only two compartments were included in the study. Upon arrival after transportation, calves were randomly allocated to two different compartments that were filled simultaneously. Calves in one of these two compartments were randomly selected to serve as negative controls, whereas calves in the other compartment were vaccinated once with commercial intranasal vaccination (Rispoval RS + Pi3 Intranasal, Zoetis) consisting of a modified live bovine respiratory syncytial virus (BRSV) and parainfluenza type 3 virus (PI-3 V). Vaccination was performed according to manufacturer’s guidelines. In total seven compartments of vaccinated calves and seven compartments of unvaccinated calves were included in the study. At the first clinical examination, sample collection and possible vaccination was performed on each calf by a veterinarian one to three days after arrival to the unit. During the study, the farmers remained blind regarding the vaccination status of the group. For the first ten days following vaccination, different overalls and boots were used in all compartments to avoid contamination of unvaccinated calves from potential nasal shedding by vaccinated calves.

### Data and sample collection

Clinical examination and sample collection was performed on all calves one to three days after arrival to the unit (mean age 19.0 days, SD 5.0) and repeated 40–44 days later (mean age 59.3 days, SD 5.3) at the time of weaning. Descriptive data of the calves enrolled in the study is presented in Table 1. All the calves were gradually weaned by rearing day 43. The calves’ weight was measured three times during the study period: At the time of arrival to the unit, at the time of second clinical examination, and right before transportation to the finishing unit. Clinical examination included measurement of body temperature, auscultation of heart and lungs, measurement of the respiratory rate and heart rate, inspection of the nose and eyes for discharge, recording of ear position, palpation and inspection of umbilicus, joints and hooves. Appearance of cough or diarrhea was also recorded. Each calf was scored 1–3 based on general appearance. The clinical examination data was used to calculate a clinical condition score and BRD score for each calf. The clinical condition score was calculated by summing up the scores of the clinical examinations (Table 3). BRD was diagnosed and scored according to the third of the three scoring systems of Love and colleagues [30] (Table 4). All clinical examinations were done by veterinarians from the research group.

**Table 3:** Summary of clinical examination scoring parameters for clinical condition score calculations

	Score		
Clinical sign	0	1	2
Rectal temperature ( °C)	< 39.2	–	≥ 39.2
Respiratory rate	< 45	≥ 45	
Respiratory sounds	Normal	Minor chances	Major chances
Ear position/Head tilt	Normal	–	Ear droop/head tilt
Nasal discharge	None	Watery discharge	Cloudy discharge
Ocular discharge	None	Watery discharge	Cloudy discharge
Umbilicus	Normal	Thickened	Inflamed
Joints	Normal	Arthritis in one joint	Polyarthritis
Bruises in joint area	None	One joint	Two or more joints
Interdigital phlegmon	None	One claw	Two or more claws
Feacal consistence	None	Loose	Watery diarrhea
Spontaneous cough	None	–	Coughing spontaneously

**Table 4:** BRD-scoring parameters for calves

Clinical sign	Level	Score
Cough	None or induced cough	0
	Spontaneous cough	2
Nasal discharge	None	0
	Any	4
Ocular discharge	None	0
	Any	2
Ear position	Normal, ear flick or head shake	0
	Ear droop or head tilt	5
Rectal temperature ( °C)	< 39.2	0
	≥ 39.2	2
Abnormal respiration^a^	Absent	0
	Present	2

^a^Auscultation was performed by using a stethoscope

Calves were categorized as BRD positive if their total score was ≥5 (modified Love et al. 2014)

After the clinical examinations were performed, a blood sample was obtained by venipuncture of the vena jugularis with a vacuum system (9 ml Z Serum Clot Activator, Vacuette, Austria). Before releasing the calf to the pen in vaccination groups, a single-use plastic nasal cannula (Zoetis) was used to administer 1 ml of vaccine to the nostril of the calf. Calves were examined in batches such that one group of vaccinated and unvaccinated calves were examined on the same day. Examinations always started with the unvaccinated group to avoid contamination from potential nasal shedding. Vaccination was not repeated on the second visit.

Throughout the study period, signs of diseases, treatments, and mortality of the calves were observed and recorded on a daily basis by the farmer. Sick calves were treated by the farmer according to the instructions of the farm’s contract veterinarian. Medication for BRD was started if two or more of the following signs were recognized: increased body temperature (> 39.7 °C), signs of depression, loss of appetite, rapid or difficult breathing. The primary medication for respiratory inflammation was a combination of parenteral oxytetracycline and parenteral non-steroidal anti-inflammatory drug (NSAID). For interdigital phlegmon or umbilical inflammation, a combination of parenteral benzylpenicillin and NSAID was used as the primary medication. The date of treatment together with all the observed signs (body temperature, signs of depression, inappetence, rapid or difficult breathing, coughing, possible eye or nasal discharge, diarrhea, interdigital phlegmon, any other signs of disease) and medicines used were precisely recorded. If the calf was seriously ill and untreatable, herd veterinarian euthanized the calf due welfare reasons. First, the calf was tranquilized with 0.2 mg/kg xylazine (Nerfasin vet 20 mg/ml, Le Vet B. V, Netherlands), and thereafter 140 mg/kg of product containing pentobarbital sodium and phenytoin sodium (Euthasol vet 400 mg/ml, Le Vet B.V). Both products injected into the jugular vein. When possible, necropsy was done to calves that died or euthanized during the study period.

### Analysis of blood samples

After the blood samples were collected, they were transported to the laboratory and stored in a Styrofoam box with cooling elements. In the laboratory, blood samples were centrifuged at 3500 rpm for 15 min to separate the serum, which was then isolated and stored at − 20 °C in 2 ml tubes (Sarstedt, Germany) until analyzed for bovine IgG and SAA concentrations. IgG of the serum samples was analyzed using a commercial ELISA kit (Bovine IgG ELISA Quantitation Set Cat. No. E10–118, Bethyl Laboratories, Inc., Montgomery, TX, USA) with dilutions 1:200000 and 1:40000 and detection ranges 1.56–100 mg/ml and 0.31–20 mg/ml, respectively. Analysis was done according to the manufacturer’s instructions. Serum SAA concentration was measured with a commercially available solid phase sandwich ELISA kit (Phase TM Range Multispecies SAA ELISA kit, Tridelta Development Ltd., Ireland) according to the manufacturer’s instructions for bovine samples. Dilutions of 1:500, 1:1000 and 1:4000 were used with detection ranges 9.4–150 μg/ml, 18.8–300 μg/ml and 75.2–1200 μg/ml, respectively.

### Statistical analysis

According to the sample size calculations, 250 controls and 250 vaccinated calves were needed to detect a difference of 10% in treatment rate between study groups (power 0.9). A *p*-value < 0.05 was considered statistically significant. Independent t-tests were used to compare the weight, age, clinical condition scores, serum IgG and SAA concentrations of the calves in different vaccination groups at the time of arrival. Descriptive data is presented in Table 1.

Linear mixed regression models were used to study the association between intranasal vaccination and daily gain of the calves during the milk-feeding period and the complete study period. Because of the interaction detected between the calf age at arrival and vaccination (Fig. 1), two separate models were used to evaluate the association between vaccination and daily gain during the complete study period. One model was used for calves 17 days or older at the time of arrival, and another model for calves younger than 17 days. Additionally, three logistic mixed regression models were used to study the association between intranasal vaccination and BRD diagnosis at the second clinical examination (0/1), treated for BRD during the study period (0/1), and death of the calf before transportation to the finishing unit (0/1). The transportation batch was used as a random factor in all models. In addition to vaccination status (vaccinated or unvaccinated), we studied several potential explanatory covariates. Nominal variables included sex (male, female) and breed of the calves (Ayrshire, Holstein, Dairy-beef crosses) and BRD diagnosis at the first clinical examination (0/1). Continuous variables included age and weight of the calves at arrival, clinical score, and serum IgG and SAA concentration at the time of the first clinical examination. Age at arrival and SAA concentration were log-transformed to achieve normal distribution (and better suitability for the models). The number of antimicrobial treatments per calf was tested in models with outcome variables such as daily gain and mortality to control the possible confounding effect on the outcomes. Moreover, the confounding effect of sex and breed was tested in all models if that variable was not already included in the model (e.g. in the case of univariate analyses). A change greater than 20% in variable coefficients or odds ratios was defined as confounding. All the biologically meaningful two-way interactions between vaccination and fixed factors were tested. All statistical analyses were performed in Stata/MP 14.1 for Windows (StataCorp LP, Texas, USA).

## Data Availability

The datasets analyzed during the current study are available from the corresponding author upon reasonable request.

## Abbreviations

BRD: Bovine respiratory disease
Coeff.: Coefficient
IgG: Immunoglobulin G
OR: Odds ratio
SAA: Serum amyloid A
